# Case Report: ERAT-based management of appendiceal abscess in early pregnancy

**DOI:** 10.3389/fsurg.2026.1797433

**Published:** 2026-05-14

**Authors:** Bowen Zhou, Yuchen Wang, Sheng Ao, Jianing Hou, Chengyi Wang, Miaomiao Yang, Yanhuan Zhong, Yangyang Xue, Chang Yan, Guoqing Lv

**Affiliations:** 1 Department of Gastrointestinal Surgery, Peking University Shenzhen Hospital, Shenzhen, China; 2 Medical School, Shenzhen University, Shenzhen, China

**Keywords:** appendiceal abscess, case report, early pregnancy, endoscopic retrograde appendicitis therapy (ERAT), percutaneous catheter drainage (PCD)

## Abstract

Acute appendicitis during pregnancy, while rare with an incidence of approximately 0.063%, represents a clinically significant condition that necessitates careful management. Current treatment strategies for appendiceal abscess include antibiotic therapy, percutaneous catheter drainage (PCD), and surgical intervention. However, surgical approaches in pregnant patients are associated with considerable obstetric risks, including miscarriage, preterm labor, and fetal demise. Moreover, Non-operative management carries a reported failure rate of up to 30%. Endoscopic retrograde appendicitis therapy (ERAT) has emerged as an advanced endoscopic technique that may provide a viable therapeutic alternative for appendiceal abscess in pregnancy. We present the case of a 24-year-old female at 3 weeks and 4 days of gestation diagnosed with a appendiceal abscess, who exhibited a suboptimal response to initial combined therapy involving ultrasound-guided PCD and ertapenem. Following adjunctive ERAT, the patient demonstrated marked clinical and laboratory improvement, with resolution of symptoms and normalization of inflammatory markers, and was subsequently discharged without any adverse pregnancy outcomes. This case suggests that ERAT may represent a safe and effective treatment option for appendiceal abscess in early pregnancy, offering a promising alternative to conventional management strategies.

## Introduction

Acute appendicitis ranks among the most common surgical conditions worldwide, with an estimated incidence of approximately 0.214% (1). Its incidence during pregnancy is relatively low, reported at around 0.063% (2). If acute appendicitis is not effectively managed, it may progress to appendiceal abscess or phlegmon in approximately 4% to 20% of cases (3). Currently, antibiotic therapy and PCD remain the mainstays of treatment for appendiceal abscess. However, a non-operative management strategy based on antibiotic therapy combined with PCD is associated with a failure rate of approximately 30%, with about 10% of patients eventually requiring surgical intervention (4).

Surgical management during pregnancy brings risks of adverse pregnancy outcomes, including miscarriage, preterm delivery, and stillbirth (5, 6). ERAT is a minimally invasive endoscopic therapeutic modality for the management acute appendicitis, providing a non-surgical treatment. As its clinical applications have expanded, this technique has evolved toward greater maturity. Recent studies have demonstrated that the success rates of ERAT in treating appendicitis are now comparable to those of open appendectomy (OA) and laparoscopic appendectomy (LA), with no significant differences in clinical outcomes (7). This case describe a first-trimester gravida presenting with a appendiceal abscess, successfully treated with a combination of ERAT and percutaneous drainage of the intra-abdominal abscess, resulting in a favorable clinical outcome, and no adverse effects happened on pregnancy.

## Case presentation

**Figure 1:**
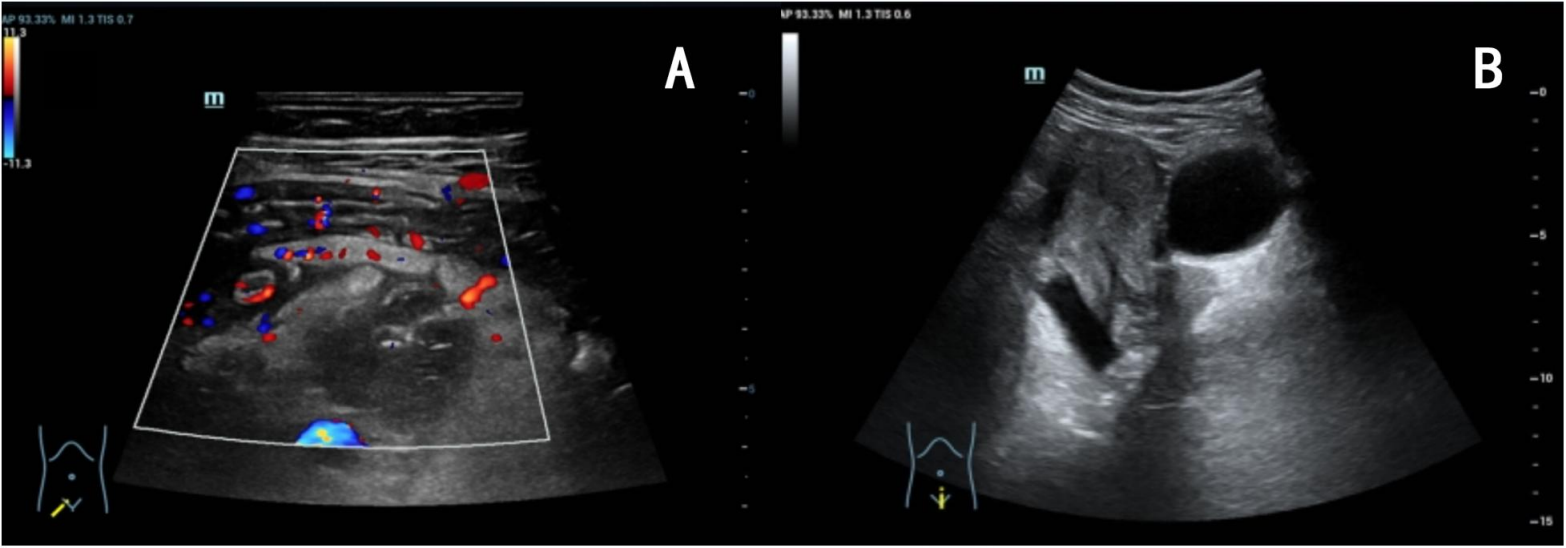
Examinations before nendoscopic retrograde appendicitis therapy. **(A)** Transabdominal ultrasonography demonstrated an appendiceal abscess in the right lower quadrant, measuring approximately 41 × 25 × 29 mm, intralesional blood flow signals are identified. **(B)** Transabdominal ultrasonography demonstrated an endometrial thickness of 9 mm with no definitive gestational sac identified within the uterine cavity.

A 24-year-old woman presented with a 25-day history of amenorrhea, 5 days of right lower quadrant abdominal pain, and fever (peak temperature, 39.0 °C). Diagnostic evaluation at our institution yielded the following findings: serum *β*-human chorionic gonadotropin (*β*-hCG) was 240 IU/L; white blood cell (WBC) count, 13.74 × 10⁹/L; interleukin-6 (IL-6), 80.7 pg/mL; and procalcitonin (PCT), 0.17 ng/mL. Ultrasonography revealed an appendiceal abscess (approximately 41 × 25 × 29 mm) in the right lower quadrant, while no definite gestational sac was identified within the uterine cavity (Figure 1). A diagnosis of appendiceal abscess in early pregnancy (gestational age, 3 weeks and 4 days) was made. The absence of a visible gestational sac was attributed to the early stage of gestation.

**Figure 2:**
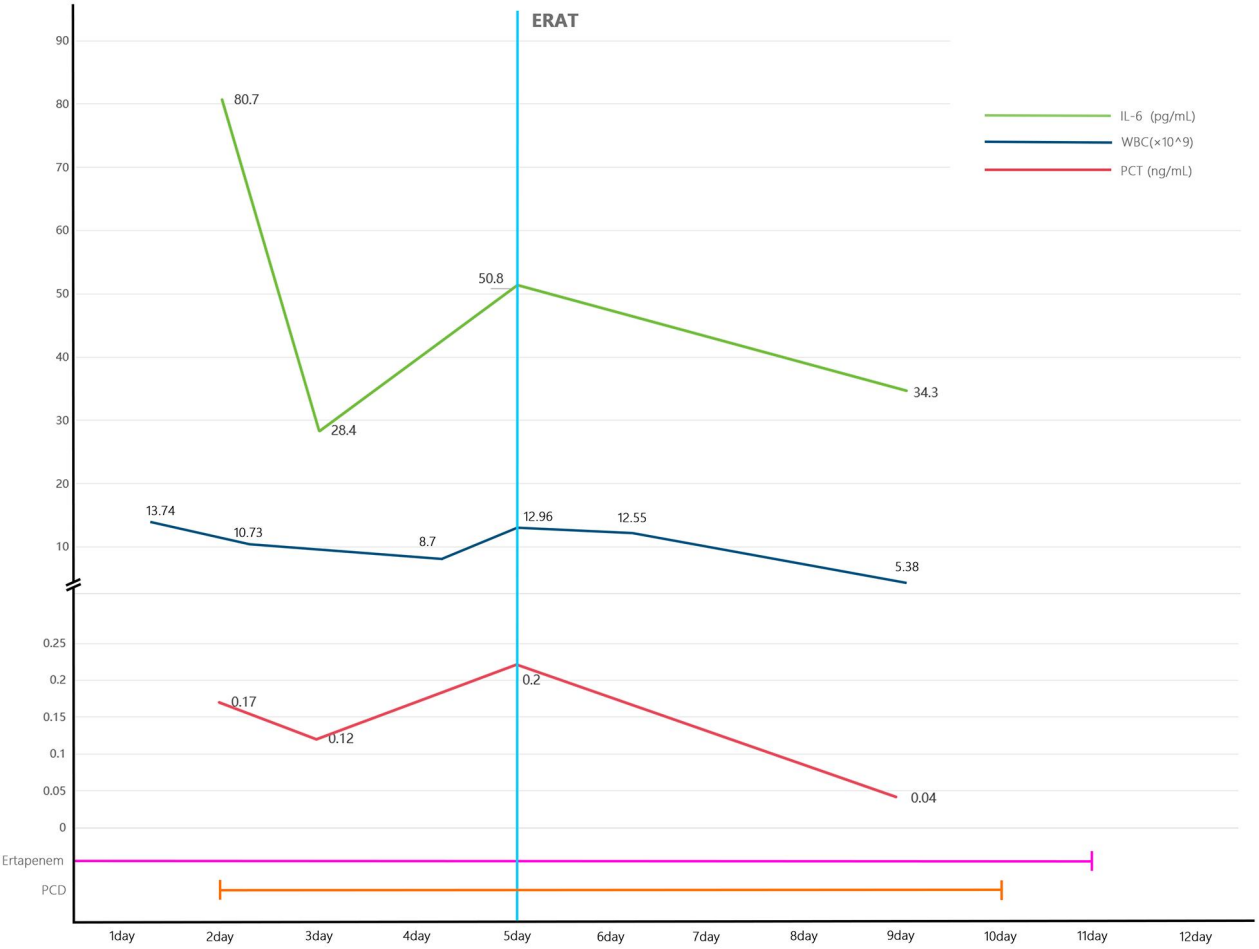
Trends in inflammatory markers and schematic of the clinical management timeline.

In an effort to manage the appendiceal abscess, intravenous antibiotic therapy with ertapenem (1 g daily) was commenced. The following day, ultrasound-guided PCD was performed, yielding 13 mL of brown, turbid fluid, which was sent for microbiological analysis. Post-procedural laboratory tests showed a reduction in inflammatory markers: WBC decreased to 8.7 × 10⁹/L, IL-6 to 50.8 pg/mL, and PCT to 0.12 ng/mL. However, the patient's abdominal pain persisted with only modest improvement, and the drainage catheter continued to output thick, yellow turbid fluid. By the third day, a progressive resurgence of inflammatory markers was observed, with WBC rising to 12.96 × 10⁹/L, IL-6 to 50.8 pg/mL, and PCT to 0.20 ng/mL (Figure 2).

Following a multidisciplinary team (MDT) consultation involving the emergency department, gastrointestinal surgery, and obstetrics and gynecology, it was concluded that the combined regimen of antibiotic therapy and PCD had produced a suboptimal response. Surgical intervention for the appendiceal abscess was considered to carry risks of miscarriage and teratogenicity. In contrast, ERAT is a well-established technique at our institution and was deemed a relatively safe option for managing the appendiceal abscess during early pregnancy. After obtaining informed consent, the patient subsequently underwent ERAT.

**Figure 3:**
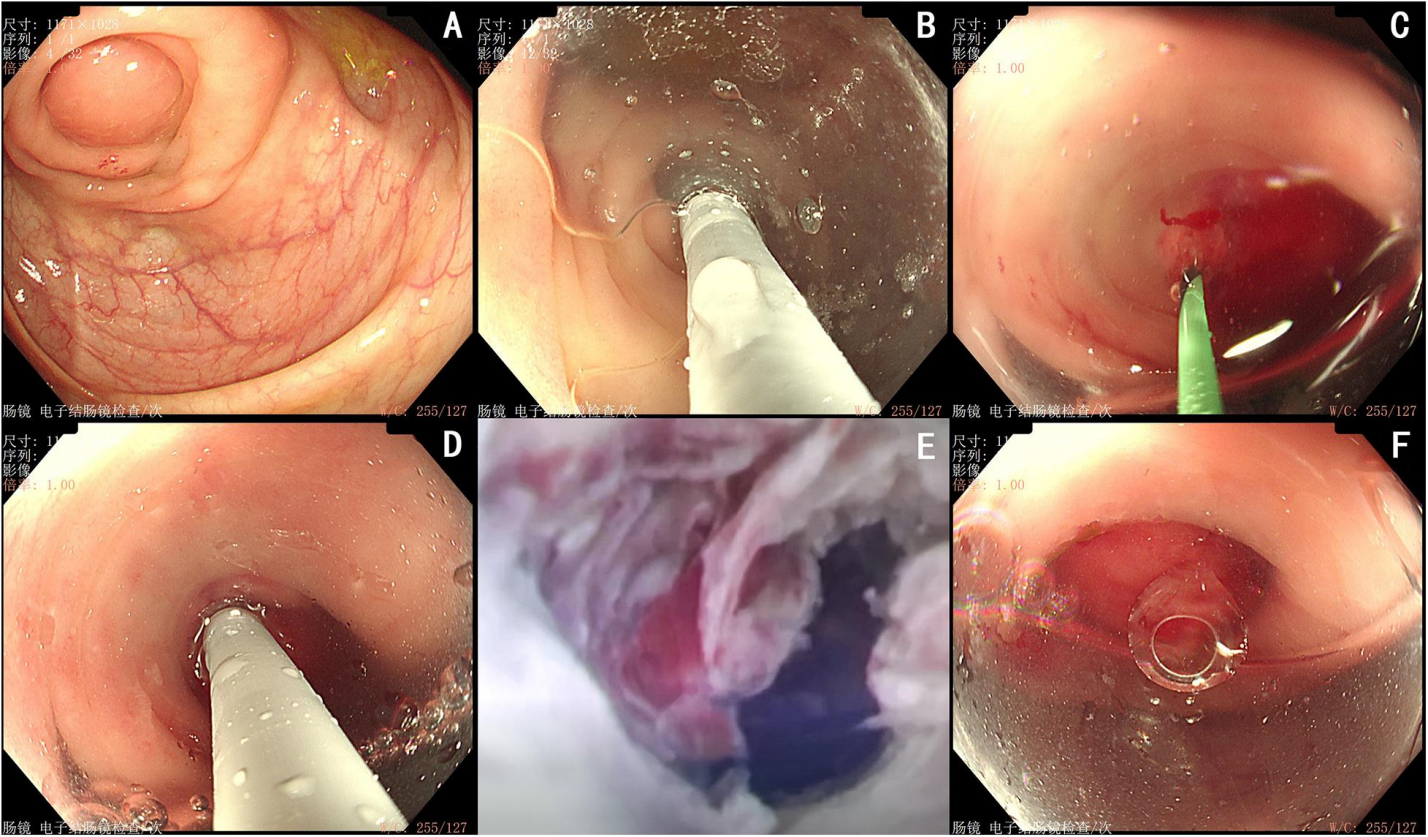
Endoscopic retrograde appendicitis therapy of periappendiceal abscess. **(A)** The endoscope reaches the opening of the appendix. **(B)** Use a clear cap to push Gerlach's flap against the appendix cavity. **(C)** Inserted guide wire. **(D)** The appendix cavity was accessed using Disposable Imaging Cathete. **(E)** The EyeMax endoscopic system revealed inflammatory edema with stenosis within the appendiceal lumen. **(F)** The appendix cavity was irrigated.

After bowel preparation, general anesthesia was induced with the safety-preferred ciprofol for the pregnant patient. Endoscopy was performed via a retrograde transanal approach. Upon reaching the cecum, the appendiceal orifice was noted to be markedly edematous, congested, and obstructed. A transparent cap was used to gently retract the Gerlach's valve away from the orifice, after which a straight-tip guidewire (0.035 in × 450 cm, Boston Scientific Corporation) was inserted, allowing drainage of a small amount of purulent secretion. Under endoscopic guidance, a disposable imaging catheter (9 Fr, outer diameter≤3.2 mm, tubing 2.9 mm, instrument channel 21.0 mm, EyeMax submicroscope system; Micro-Tech, Nanjing, China) was successfully positioned. Within the appendiceal lumen, inflammatory edema with associated stenosis was visualized. The cavity was then thoroughly irrigated, completing the procedure (Figure 3).

Following treatment with antibiotic therapy, PCD, and subsequent ERAT, the patient became afebrile and experienced complete resolution of abdominal pain. Inflammatory parameters improved significantly, with a white blood cell count of 5.38 × 10⁹/L, a procalcitonin level of 0.04 ng/mL, and an interleukin-6 level of 34.3 pg/mL (Figure 2). No vaginal bleeding was observed during the hospitalization. Drainage output remained negligible for four consecutive days, enabling uneventful removal of the catheter and subsequent discharge. Microbiological analysis of the drained fluid identified Escherichia coliand, with susceptibility to ertapenem confirmed.

**Figure 4:**
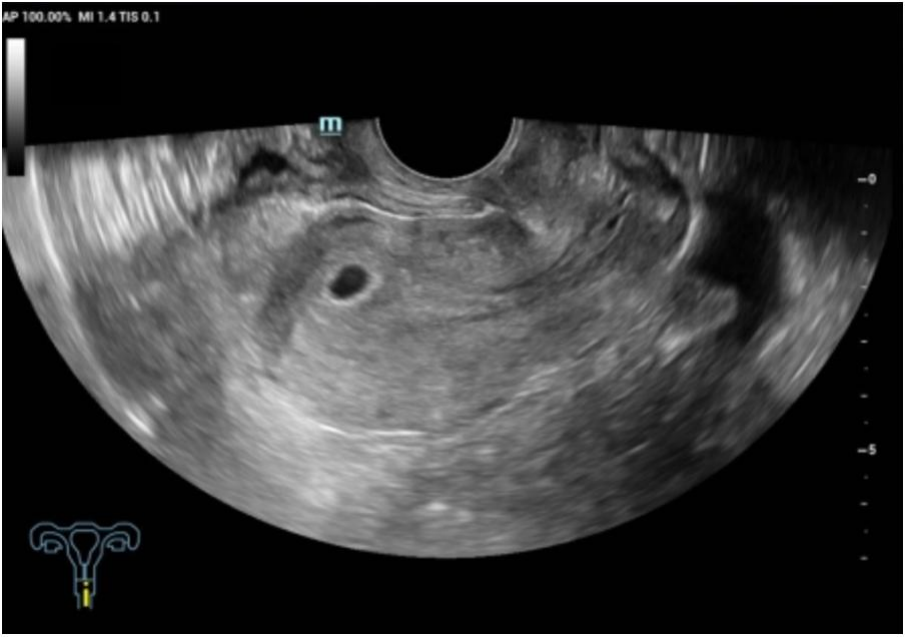
Five days after the operation, transvaginal color Doppler ultrasound showed the echo of the gestational sac in the uterine cavity, and the size was about 10 × 6 × 7 mm.

5 days after ERAT, transvaginal ultrasound confirmed a viable ongoing intrauterine pregnancy (Figure 4). Serial outpatient follow-up through 24 weeks of gestation demonstrated no obstetric complications. Thereafter, the patient was lost to follow-up, and the final obstetric outcome remains unknown.

## Discussion

ERAT was initially inspired by Endoscopic Retrograde Cholangio-pancreatography (ERCP) and was first applied to the treatment of appendicitis by Chinese doctor Liu Bingrong in 2009 (8, 9). This technique involves acessing the cecum via colonoscopy, visualizing the appendiceal opening, and performing procedures such as catheterization, lavage, fecal stone removal, drainage tube or stent placement under intervention or endoscopic guidance (10).

Traditional nonoperative management of acute appendicitis primarily based on antibiotic therapy and PCD, carries a non-negligible risk of treatment failure, especially in the presence of a complex condition such as an appendiceal abscess (11, 12). For appendicitis and periappendiceal abscess during pregnancy, selecting the optimal treatment requires a careful integration of the benefits of antibiotic therapy, PCD, and surgery. A tailored and individualized approach is essential to ensure the safety of both the mother and the fetus. PCD aims to alleviate localized inflammation through abscess cavity puncture and drainage. However, percutaneous drainage presents several limitations. The puncture procedure carries potential risks of iatrogenic injury to adjacent intestinal structures and vasculature. Furthermore, inadequate drainage resulting from extensive abscess dimensions, multiloculated configurations, or catheter obstruction may compromise effective infection control (13). Notably, clinical evidence indicates that approximately 25.4% of patients undergoing percutaneous drainage ultimately require surgical intervention as definitive treatment (14).

The application of ERAT in the treatment of appendicitis precisely addresses the inherent limitations of conventional therapeutic approaches. ERAT has demonstrated successful application across a spectrum of appendicitis types. For uncomplicated appendicitis, it can achieve noninferior efficacy compared to antibiotic therapy and surgery (15). Furthermore, its use has been extended to manage complex cases, including chronic appendicitis, appendiceal perforation, appendicitis with fecalith, appendiceal abscess, and stump appendicitis (16–18). Therefore, incorporating ERAT into a comprehensive treatment strategy could yield favorable outcomes for patients with acute appendicitis, thereby avoiding surgery and its associated risks.

In addition, the negative appendectomy rate (NAR) has remained a clinical dilemma confronting surgeons in practice. Studies have reported an incidence of negative appendectomy of 13% in patients undergoing surgery for appendicitis (13). ERAT is not only a therapeutic approach but also a diagnostic tool for gastrointestinal diseases. When acute appendicitis is suspected but cannot be definitively diagnosed based on clinical symptoms, imaging findings, or inflammatory markers, ERAT offers a novel diagnostic and therapeutic alternative. ERAT involves advancing a colonoscope to the cecum to directly visualize the appendiceal orifice. This allows for the detection of inflammatory signs such as edema and purulent discharge, confirms the diagnosis of appendicitis, rules out other concurrent gastrointestinal inflammatory conditions, and helps to avoid negative appendectomy.

This study reports a case of an early-pregnancy appendiceal abscess managed with a combination of ERAT and PCD, alongside systemic antibiotic therapy with ertapenem. This integrated approach successfully preserved the intrauterine pregnancy without adverse obstetric outcomes such as miscarriage. Notably, even without placing an intraoperative drainage tube, the ERAT procedure achieved effective internal drainage through direct endoscopic lavage of the abscess core. This mechanism proved pivotal for infection control. When combined with percutaneous drainage, this dual-modality strategy facilitates rapid containment of the infectious focus. The presented treatment offers an innovative, minimally invasive alternative for managing intra-abdominal infections secondary to appendiceal abscess, thereby avoiding the need for surgical intervention. These findings provide clinical evidence supporting the application of ERAT in complex appendicitis, particularly during the first trimester in cases complicated by appendiceal abscess formation.

Despite its widespread application across appendicitis subtypes, ERAT exhibits inherent limitations. First, surgical manipulation carries the risk of mechanically occluding the appendiceal lumen, thereby compromising drainage efficacy. Second, inadequate formation of the abscess capsule may result in iatrogenic accumulation of intraperitoneal fluid. Third, progression of infection beyond the scope of endoscopic control renders surgical intervention indispensable. In addition, research on ERAT remains limited, resulting in a lack of widely accepted consensus on its indications and standardized protocols. This evidence gap has impeded the technique's global adoption. To address this, establishing formal standards to define clinical indications, optimize protocols, and ensure safety is crucial to overcoming current limitations and advancing the technique.

Finally, several limitations of this study should be acknowledged. These include its single-case design, short-term follow-up, and the inherent constraints on generalizability to the broader pregnant population. Future studies with larger sample sizes and longer follow-up periods are warranted to further validate these preliminary findings.

## Conclusion

This clinical application demonstrates that ERAT is a safe and effective treatment for appendiceal abscesses in early pregnancy, may present a highly promising therapeutic approach for appendicitis in pregnancy.

## Data Availability

The original contributions presented in the study are included in the article/Supplementary Material, further inquiries can be directed to the corresponding authors.
